# Microplastic Contamination: A Rising Environmental Crisis With Potential Oncogenic Implications

**DOI:** 10.7759/cureus.85191

**Published:** 2025-06-01

**Authors:** Muhammad Imran

**Affiliations:** 1 Biochemistry Section, Institute of Chemical Sciences, Faculty of Life and Environmental Sciences, University of Peshawar, Peshawar, PAK

**Keywords:** cancer, carcinogen, environmental pollution, microplastics, oncology, public health

## Abstract

Microplastics have emerged as pervasive environmental contaminants detected in air, water, food, and even human tissues such as blood and placenta. These particles are now recognized as potential health hazards to humans. Due to their high surface area and ability to adsorb toxic substances, microplastics can act as vectors for heavy metals and persistent organic pollutants, many of which are established carcinogens. Evidence from animal models links microplastic exposure to oxidative stress, inflammation, immune disruption, and tissue damage, mechanisms implicated in cancer pathogenesis. Although direct causal links in humans need investigation, microplastics have been associated with occupational cancer risks and shown to interfere with cellular and metabolic processes. This emerging health threat underscores the urgent need for regulatory oversight, targeted research, and comprehensive public health strategies to mitigate exposure and assess long-term oncogenic implications.

## Editorial

Introduction

The 21st century has witnessed remarkable technological advancements, yet it has also ushered in new environmental threats. One such pressing concern is the widespread contamination of ecosystems by microplastics. Microplastics, defined as synthetic polymer particles measuring less than 5 mm in diameter, have become pervasive environmental contaminants. They have been identified across a broad range of ecological niches, including marine sediments, terrestrial surfaces, and atmospheric samples, and within consumables such as water and food products. While their ecological impact is now well documented, a growing body of research suggests that microplastics may also pose serious risks to human health, e.g., cancer development.

Microplastics are either primary (manufactured at a microscopic size, e.g., in cosmetics or industrial abrasives) or secondary (resulting from the degradation of larger plastic items). They have been detected in marine organisms, freshwater sources, soil, air, table salt, bottled water, human blood, and placenta. Plastic polymers' high durability and chemical stability, once considered a boon, now threaten biodiversity and public health due to their persistence in the environment. Humans are exposed to microplastics through ingestion, inhalation, and dermal contact. Of particular concern are the potential long-term health outcomes, including chronic inflammation and carcinogenesis.

Mechanistic insights and research gaps

Microplastics can act as physical and chemical stressors in biological systems. When ingested or inhaled, they may trigger oxidative stress, chronic inflammation, immune dysfunction, and cellular toxicity [1]. Chronic oxidative stress, for instance, can damage DNA, proteins, and lipids, initiating mutagenesis and impairing genomic stability. Simultaneously, unresolved inflammation contributes to a pro-tumorigenic microenvironment through sustained cytokine release and immune evasion [1-3]. All these are well-established hallmarks of carcinogenesis. Moreover, microplastics have a high surface-area-to-volume ratio and hydrophobic surfaces, which allow them to adsorb and transport hazardous substances such as polycyclic aromatic hydrocarbons, heavy metals, and persistent organic pollutants. These substances are known mutagens and carcinogens. Microplastics act not only as contaminants themselves but also as vectors for other toxins, potentially increasing their bioavailability and toxicity. In animal models, such as mice and zebrafish, exposure to polystyrene microplastics has resulted in intestinal barrier dysfunction, hepatic fibrosis, altered metabolic profiles, and reproductive toxicity, which collectively suggest systemic physiological stress conducive to malignant transformation. Furthermore, microplastics have been shown to alter the gut microbiota composition, induce epithelial dysregulation, and promote pro-inflammatory signaling, all of which are implicated in the pathogenesis of colorectal and hepatocellular carcinomas [2,3]. Microplastics may interact with therapeutic drugs, particularly chemotherapeutics, by altering their absorption, distribution, metabolism, and excretion (ADME) profiles. Their large surface area and chemical affinity allow microplastics to adsorb drug molecules, potentially reducing bioavailability, modifying pharmacokinetics, or even causing drug resistance [4]. Additionally, microplastics may influence hepatic enzyme activity and gut barrier integrity, both of which are critical in drug metabolism and efficacy [1,3]. Such interactions raise concerns regarding variable drug responses, particularly in populations with chronic microplastic exposure. Further in vivo studies are essential to clarify these risks and their clinical significance.

While direct evidence of microplastics causing cancer in humans remains limited due to the novelty and complexity of the subject, recent studies offer alarming indications. For instance, a study by Horvatits et al. found microplastics in the liver tissue of patients with liver cirrhosis, implying systemic circulation and organ-level accumulation [5]. Inflammation in such vital organs is a known precursor to hepatocellular carcinoma and other cancers. Moreover, microplastic particles have been identified in human placentas, raising concerns about developmental toxicity and transgenerational health impacts, including increased cancer susceptibility in offspring. A preclinical study reported that chronic exposure to polystyrene microplastics in mice caused gut microbiota dysbiosis, inflammation, and epithelial dysregulation, conditions that heighten the risk of colorectal cancer [3]. Industries that heavily utilize plastics, such as manufacturing and textiles, have also seen reports of increased cancer incidences. Workers exposed to airborne micro- and nanoplastics may face heightened risks of respiratory diseases, including nasopharyngeal carcinoma and lung cancer. While not definitively causal, these correlations warrant rigorous epidemiological investigations. This parallels asbestos, once considered harmless, which later became notorious for its carcinogenicity.

Although a growing body of evidence indicates the potential adverse health impacts of microplastic exposure, comprehensive regulatory oversight remains absent in most national and international policy frameworks, particularly in low-income and developing countries. Existing food safety and water quality guidelines seldom account for the presence of microscopic synthetic polymer particles, thereby exposing a critical gap in current public health safeguards. A major impediment to regulatory progress is the absence of standardized protocols for the detection, characterization, and quantification of microplastics in both environmental and biological matrices. This methodological variability significantly limits the ability to establish consistent exposure benchmarks and hampers the reliability of toxicological risk assessments. Moreover, the majority of toxicological investigations to date have predominantly focused on larger plastic particles (meso- and macroplastics), while smaller microplastics, particularly those within the nano- to micrometer scale, may exhibit enhanced biological reactivity and cellular interactions due to their elevated surface area-to-volume ratio and increased potential for bioavailability. These smaller particles are more likely to cross biological barriers, accumulate in tissues, and interact with cellular components. Long-term, low-dose exposures, which are more reflective of real-world human scenarios, remain poorly studied, especially regarding chronic diseases such as cancer. Moreover, the size, shape, polymer type, and surface chemistry of microplastics can all influence their biological impact, complicating risk modeling. There is a growing consensus on the critical need for well-designed longitudinal cohort studies, the implementation of standardized analytical methodologies, and the promotion of interdisciplinary collaboration. These efforts are essential to comprehensively assess and elucidate the potential carcinogenic effects of microplastic exposure in humans.

Call to action: toward a preventive approach

Given the plausible oncogenic mechanisms and preliminary findings, the precautionary principle must guide our response. Immediate steps should focus on multiple fronts. Governments should revise environmental and health safety standards to incorporate systematic monitoring of microplastics in air, water, and food systems. Simultaneously, there is a pressing need for increased investment in research, particularly in mechanistic, epidemiological, and longitudinal studies, to validate the potential cancer risks and to identify populations that may be especially vulnerable. Public engagement is equally critical; raising awareness can help promote behavior change, such as reducing plastic use, supporting sustainable alternatives, and minimizing overall plastic consumption to lower individual exposure. From a healthcare perspective, targeted medical surveillance is warranted for high-risk groups, including industrial workers and communities residing near plastic production facilities. Finally, because microplastic contamination is a transboundary issue similar to climate change, it demands coordinated international cooperation across policy, scientific, and technological domains to ensure effective and sustained mitigation.

Conclusion

Microplastics represent an invisible yet pervasive environmental hazard. While the current body of evidence on their carcinogenicity is not yet conclusive, the mechanisms linking microplastic exposure to cancer are biologically plausible and increasingly supported by experimental findings. It is imperative not to repeat past mistakes, like with tobacco, asbestos, or lead, by waiting for irrefutable evidence before taking preventive action. The stakes are too high, and the cost of inaction could devastate public health and planetary well-being.
